# An Essay on the Disorders of Old Age, and on the Means for Prolonging Human Life

**Published:** 1818-02

**Authors:** 


*An Essay on the Disorders of Old Age, and on the Means for prolonging Human Life.* By ANTHONY CARLISLE, F. R. S. F. S. A. F. L. S. *with Titles out of number; and a white, red, and black title page.* 8*vo. pp.* 103. 1817.

WHEN we read in the newspapers of this Essay on Longevity, " to be printed in *large* characters," we felt something like a presentiment that there would be *little* of real value therein ; but when our bookseller handed us a volume, whose title page resembled Harlequin's jacket in variety of colours, and whose body was almost as thin as Harlequin's lath, we were pretty well confirmed in our conjectures. An arch and acute brother critic, whose skill in biblical physiognomy cannot be questioned, has very justly observed, that a *red* face and *emaciated* body are strong indications of internal unsoundness. Had he looked more narrowly into the features of this book's face, he would probably have concluded, that the black and white streaks interspersed among the red, augured much of want of *vital energy* in the central organ, as well as great *derangement* in the capillary circulation ! Maskel Hall, in his Principles of Diagnosis, would, we are sure, predict, in this case, a speedy *mortification*, if not a total stop to the *circulation*.

But metaphor aside. PROFESSOR Carlisle dedicates his work to the College of Surgeons, " from a hope, that such proceeding will tend *most effectually* to promote the healing art." He also submits these his " professional sentiments to their competent and equitable judgment." Now we should have expected, in this case, something like *medical* facts or *medical* reasonings ; but no, " the present Essay is addressed principally to persons already advanced in years," and does not contain a single particle of useful knowledge from Alpha to Omega ! We are sincere when we assert it as our firm belief, that the feeble efforts of our author could not have taken a more effectual mode of lowering and degrading, in the eyes of the public, a liberal and learned profession. What will be thought of that College, whose " Professor of Surgery and Anatomy" has sent forth, as his " *deliberate thoughts*," and dedicated to them too, a volume which would not be owned by the most ignorant tyro or superannuated driveller in the profession !

No person, it is true, can, for a moment, doubt the *design* of this Essay ;—

"At this critical period [the seventh age of Shakspeare] it behoves the tenant to keep a strict watch over *needful repairs;* and if he have *a skilful medical architect* (aye there's the rub!) he may then obtain useful information respecting the best materials for *keeping his building together*, and on the fittest *cements* and supports to protect it against approaching storms." p. 8.

This is a specimen of that *medical elegance* which suits a work dedicated to the College of Surgeons. The following passage contains a most *novel* and important *truth*, which we believe no man, woman, or child, on the face of the Globe, will be disposed to contradict. Professor Carlisle has never known "any *serious* affections of the stomach, bowels, or liver, without the precedence of some morbid visitation, such as head-ache, flatulency, acidity, or *local pain.*" p. 10. But at page 18, the Professor takes a higher flight; he professes [it is always *I profess*] himself doubtful respecting such things as *idiosyncracies* of constitution, and gives a most luminous and ingenious solution of the difficulty—"I apprehend, that a *deeper* scrutiny will assign those apparent incongruities to the *variable degrees of power in the living organs.*" 18.

Our ingenious and witty contemporary,* represents the most eligible mode of making toast and water, and the best way of boiling cabbage, as the principal novelties in our author's work. But surely he must have overlooked the two tables at pages 40 and 44, where the octogenarian hypochondriac is furnished with the exact doses of Henry's calcined magnesia, carbonate of potash, prepared chalk, or liquid ammonia, to be taken after each glass of Port wine, Vidonia, Sherry, London Porter, or brewer's table beer,—to neutralize the acids of these beverages in their stomachs! At p. 44 we have a table of doses of the above mentioned alkalis to be taken after each "common sized lemon, sweet orange, and nonpareil apple."

Professor Carlisle is satisfied that acids act not only on the stomach, but on the whole body; and why?—"because I have, says the Professor, repeatedly felt a gouty pain and swelling in the large joint of the *great toe while drinking half a pint of Claret!!*" What a deep and logical conclusion! The readers of the "Essay on Old Age" will be at no loss where to go, when they peruse the following passage.

---

* Annals of Medicine and Surgery, No. 8.

"It is both an act of justice *to the public and to myself* to add, that *my* practice, when it has *come in contact* with gouty persons, has been governed by the preceding views, and attended with *unvarying beneficial results.*" p. 47.

The explanations given by Currie and others of the *modus operandi* of aqueous effusions on the human surface, sink into insignificance when compared with those given by the Professor.

"Ablutions of water have a constricting influence upon the living fibres, independent of temperature; an effect, perhaps, similar to that of *crimping fish.*" p. 61.

But it is time to take leave of this performance, on which we should not have spent so many words, did we not, within these few days, see the learned secretary of a medical society, completely *taken in*, by ordering it among other *medical* books, though not one of the society afterwards took the trouble of reading five pages of the work. In one line, the Professor's volume is, like those for whom its large type and speckled title page are designed,

Sans *eyes*, sans teeth, sans *taste*,—sans every thing!

